# METTL3-mediated m6A modification regulates D-galactose-induced skin fibroblast senescence through miR-208a-5p

**DOI:** 10.3389/fimmu.2025.1577783

**Published:** 2025-06-06

**Authors:** Gaoxiang Huang, Sainan Sun, Mingde Liao, Jing Wang, Qing Yan, Jing Li, Yi Meng, Qi Wang, Zhen Guo, Jiyong Tan, Jing Li

**Affiliations:** ^1^ School of Basic Medical, Guangxi Medical University, Nanning, Guangxi, China; ^2^ Zhanjiang Institute of Clinical Medicine, Central People’s Hospital of Zhanjiang, Zhanjiang, China; ^3^ Key Laboratory of Longevity and Aging-Related Diseases of Chinese Ministry of Education, Guangxi Medical University, Nanning, Guangxi, China; ^4^ Department of Plastic & Cosmetic Surgery, The First Affiliated Hospital of Guangxi Medical University, Nanning, Guangxi, China; ^5^ College of Pharmacy, Guangxi Medical University, Nanning, Guangxi, China; ^6^ Key Laboratory of Basic Research on Regional Diseases (Guangxi Medical University), Education Department of Guangxi Zhuang Autonomous Region, Nanning, Guangxi, China

**Keywords:** aging, M6A, METTL3, miR-208a-5p, senescence

## Abstract

**Introduction:**

As the most abundant epitranscriptomic modification, N6-methyladenosine (m6A) critically influences aging and age-related pathologies. However, its regulatory interplay with microRNAs (miRNAs) in skin aging remains poorly defined.

**Methods:**

Aging phenotypes were recapitulated using D-galactose (D-gal)-induced senescence models in mouse skin fibroblasts (MSFs) and mice. Interventions included METTL3 overexpression/knockdown, miR-208a-5p mimic/inhibitor transfection, and pharmacological mitophagy induction (GSK). Molecular analyses assessed m⁶A dynamics, gene regulation, and mitochondrial function.

**Results:**

In D-gal-induced aging models, global RNA hypomethylation and reduced METTL3 expression were observed, while METTL3 overexpression attenuated cellular senescence. Mechanistically, METTL3 depletion elevated miR-208a-5p levels via YTHDF2-mediated m⁶A recognition, establishing epitranscriptional control. This upregulated miR-208a-5p directly targeted the 3'-UTR of OPA1 (optic atrophy type 1), suppressing mitophagic activity. Critically, senescent phenotypes induced by METTL3 knockdown or miR-208a-5p mimicry were reversed by pharmacological mitophagy induction (GSK), confirming mitochondrial homeostasis as the pathway's functional nexus.

**Discussion:**

These results establish an m6A-dependent METTL3/miR-208a-5p/OPA1 axis that regulates mitophagy and skin aging. Pharmacological rescue of mitophagy highlights this pathway's therapeutic relevance for age-related dermatopathology.

## Introduction

Aging is an evolutionarily conserved biological process defined by hallmark features including telomere attrition, genomic instability, proteostatic collapse, metabolic dysregulation, epigenetic drift, mitochondrial dysfunction, cellular senescence, stem cell depletion, and disrupted intercellular communication (1–3). As a sentinel organ, the skin exhibits age-related physiological decline marked by progressive loss of cellular functionality and structural integrity, serving as both a biomarker and driver of systemic aging (4–6). Cutaneous aging, shaped by intrinsic molecular programs and extrinsic environmental stressors, correlates with pathological sequelae ranging from immune compromise to carcinogenesis. Central to this process is the collapse of collagen homeostasis—a critical determinant of dermal architecture Dermal fibroblasts, the principal collagen - producing cells, undergo SASP activation, leading to impaired ECM synthesis and the release of pro - inflammatory factors that exacerbate inflammation and impact immune function, contributing to skin aging. This senescence-driven collagen deficiency underlies hallmark aging phenotypes: loss of elasticity, wrinkle formation, and mechanical failure of the dermal scaffold (7, 8).

N6-methyladenosine (m6A), the most abundant internal RNA modification in eukaryotes, is dynamically regulated by a tripartite molecular machinery: writers (METTL3/METTL14/WTAP methyltransferase) (9–11), erasers (ALKBH5/FTO demethylases) (12, 13), and readers (YTHDF2/HNRNPA2B1) (14). This epitranscriptomic mark governs RNA metabolism—splicing, stability, translation, and nuclear-cytoplasmic shuttling—with demonstrated roles in neurodegeneration (15), metabolic disorders (16), and oncogenesis (17). Emerging studies implicate m6A dysregulation in aging trajectories, including METTL3/14 redistribution driving SASP activation via m6A-independent transcriptional mechanisms (18)and diminished m6A levels accelerating senescence in human mesenchymal stem cells (19).

MicroRNAs (miRNAs), small non-coding RNAs (~22 nt), post-transcriptionally silence gene expression through 3′-untranslated region (3′-UTR) targeting, emerging as master regulators of aging pathways (20). These molecules exhibit pleiotropic effects across tissues—for instance, miR-302b-3p modulates skin fibroblast senescence via direct suppression of JNK2 (21). Intriguingly, m6A modifications intersect with miRNA biology, regulating miRNA biogenesis, stability, and target engagement across diverse aging contexts (22–24).

Despite these advances, the functional interplay between m6A and miRNA networks in skin aging remains uncharted. Therefore, the primary objective of this study was to investigate the role of METTL3-mediated m6A modification in regulating D-galactose-induced senescence in skin fibroblasts and to elucidate the mechanistic involvement of miR-208a-5p in this process. Here, we delineate a METTL3-m6A-YTHDF2 axis that governs miR-208a-5p biogenesis in mouse skin fibroblasts (MSFs). METTL3 depletion reduces m6A deposition, enabling YTHDF2-mediated stabilization of miR-208a-5p, which targets OPA1 to disrupt mitophagy and potentiate senescence. Our findings unveil novel therapeutic targets for mitigating skin aging and its associated pathologies.

## Materials and methods

### Animals and treatment

**Figure 1:**
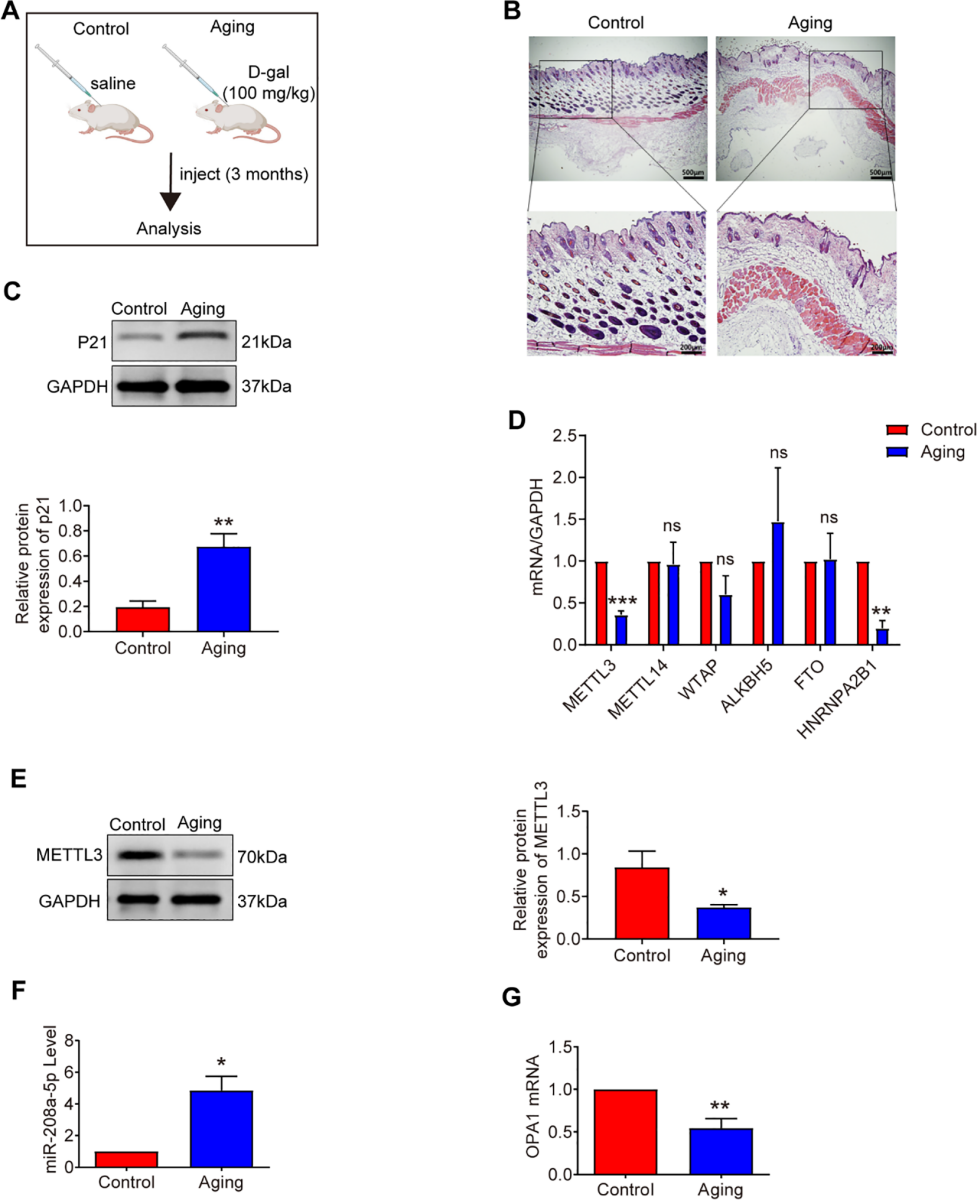
Analysis of METTL3, miR-208a-5p and OPA1 in aging skin. **(A)** Experimental design overview. **(B)** Hematoxylin and eosin (H&E) staining of mouse skin tissue after daily subcutaneous injections of D-galactose for three months (n=10). Scale bars: 500μm (overview), 200μm (magnified view). **(C)** Western blot analysis of P21 expression in aging mice (n=3). **(D)** qRT-PCR measurement of m6A-modified mRNA levels in aging skin (n=5). **(E)** Western blot analysis of METTL3 expression in aging skin (n=3). **(F)** qRT-PCR quantification of miR-208a-5p levels in aging skin (n=3). **(G)** qRT-PCR analysis of OPA1 mRNA expression in aging skin (n=3). All data are presented as mean ± SEM. *p < 0.05, **p < 0.01, ***p < 0.001, ns was considered statistically insignificant. D-gal: D-galactose. **Figure 1A** was created with BioRender.com.

Chronic D-galactose (D-gal) exposure recapitulates systemic aging in rodents, including cutaneous manifestations (25). Six-week-old female BALB/c mice (Animal Center of Guangxi Medical University, Nanning, China) were randomized into control (saline, n=10) and aging (100 mg/kg D-gal, n=10) groups. After 1-week acclimatization, aging cohort mice received daily subcutaneous D-gal injections for 3 months (**Figure 1A**). Post-treatment, mice were euthanized by cervical dislocation, and skin tissues were either processed immediately or stored at -80°C. All protocols adhered to Guangxi Medical University Animal Ethics Committee guidelines.

### Cell culture and treatment

Primary mouse skin fibroblasts (MSFs) were isolated from dorsal skin of 5-day-old neonates using established protocols (21). For senescence induction, passage 3–5 MSFs at 50% confluence were treated with 20 g/L D-gal-supplemented medium for 48 h. All experiments were performed with three independent biological replicates, defined as cells isolated from three distinct mice (each replicate derived from a separate animal).

### Transfection

The mimic and inhibitor oligonucleotides for miR-208a-5p were synthesized by RiboBio (Guangzhou, China), as detailed in **Table 1**. A mimic negative control (NC-mimic) and an inhibitor negative control (NC-inhibitor), both sourced from RiboBio (Guangzhou, China), were utilized as the negative controls (NC). Transfections were performed using the lipid carrier Lipofectamine 2000 (Invitrogen). Furthermore, siRNAs targeting METTL3, YTHDF2, and OPA1 (RiboBio, Guangzhou, China), as listed in **Table 1**, were transfected using Lipofectamine RNAiMAX (Invitrogen). MSFs were seeded in 6-well plates at a density corresponding to 50% confluence and subsequently infected with a METTL3 overexpression lentivirus (METTL3-OE) or a negative control lentivirus (NC-OE).

**Table 1:** The sequences of the siRNA, miRNA mimic, and miRNA inhibitor constructs.

Name	Sequences
METTL3-siRNA	TAATGTCCCATACGGTAGC
YTHDF2-siRNA	CTAGAGAACAACGAGAATA
OPA1-siRNA	GCTTACATGCAGAATCCTA
miR-208a-5p mimic	GAGCUUUUGGCCCGGGUUAUAC
miR-208a-5p inhibitor	GUAUAACCCGGGCCAAAAGCUC

### Senescence-associated β-galactosidase staining

Following the treatment, MSFs were gently washed three times with phosphate-buffered saline (PBS) to remove any residual medium or reagents. Subsequently, the cells were stained using a freshly prepared SA-β-Gal staining solution, strictly adhering to the manufacturer’s protocol (Beyotime, China). After staining, the cells were examined under a microscope (Leica, Germany) to assess the staining pattern and intensity, which is indicative of senescence-associated β-galactosidase (SA-β-Gal) activity.

### 5-Ethynyl-2’-deoxyuridine assay

The EdU assay was performed utilizing the BeyoClick™ EdU Cell Proliferation Kit (Beyotime, China), with Alexa Fluor 594 as the detection fluorophore. For nuclear staining, a Hoechst 33342 staining solution was obtained from Beyotime. Following the staining procedures and subsequent washing steps, the cells were examined under an inverted fluorescence microscope (Leica, Germany) to assess cell proliferation and nuclear morphology.

### Scratch assay

The Scratch assay protocol was in accordance with reference (26). To ensure consistent imaging, create reference markings near the scratch. Once the markings are in place, position the plate under a microscope (Leica, Germany) to capture the initial image of the scratch. After incubation, realign the reference point and take a second image. For further quantitative analysis, the acquired images for each sample can be analyzed using Image Pro-Plus software.

### Histological analysis

Skin tissues were collected and fixed with 4% paraformaldehyde for one week. Then, the tissues were dehydrated using a gradient ethanol solution and immersed in paraffin for two hours. After embedding, the tissues were cut to make 4 μm-thick serial sections. All the sections were prepared for hematoxylin-eosin staining (H&E; Solarbio, China). The stained specimens were examined under a light microscope (Leica, Germany) to evaluate the histological features.

### Mitophagy detection

After treatment, the mitophagy of MSFs was assessed using the Mitophagy Detection Kit (Dojindo Molecular Technologies, Inc., Japan), following the manufacturer’s instructions. To induce mitophagy, GSK2578215A (GSK) (Beyotime, China) was employed (27). After washing the MSFs twice with a serum-free medium, the cells were observed under a fluorescence microscope (Leica, Germany).

### RNA isolation and quantitative RT-PCR

Total RNA was extracted from skin tissues and MSFs using TRIzol reagent (Invitrogen). Reverse transcription was performed using the ReverAid First Strand cDNA Synthesis Kit (Thermo, United States) for mRNA and the Mir‐X miRNA First Strand Synthesis Kit (Clontech, Dalian, China) for miRNA. The resulting cDNA products were used as templates for quantitative real-time PCR (qRT-PCR) amplification, which was carried out using the Power SYBR Green PCR Master Mix (ABI, UK). The miRNA 3′ Primer and U6 were provided in the respective kits. For normalization, GAPDH was used as the control for mRNA quantification, and U6 was used for miRNA quantification. The relative expression levels of mRNA or miRNA were calculated using the 2^–ΔΔCT^ method. The sequences of the primers used in this study are listed in **Table 2**.

**Table 2:** Primer sequences for genes and miRNAs.

primer name		Primer sequences
GAPDH	Forward	GGTTGTCTCCTGCGACTTCA
	Reverse	TGGTCCAGGGTTTCTTACTCC
METTL3	Forward	CGCTGCCTCCGATGTTGATCTG
	Reverse	TCTCCTGACTGACCTTCTTGCTCTG
METTL14	Forward	TCACCTCCTCCCAAGTCCAAGTC
	Reverse	CCCTAAAGCCACCTCTCTCTCCTC
WTAP	Forward	ACGCAGGGAGAACATTCTTGTCATG
	Reverse	TCGGCTGCTGAACTTGCTTGAG
ALKBH5	Forward	CGGGACCACCAAGCGGAAATAC
	Reverse	CCTCTTCCTCCTTCTGCAACTGATG
FTO	Forward	CTCACAGCCTCGGTTTAGTTCCAC
	Reverse	CGTCGCCATCGTCTGAGTCATTG
HNRNPA2B1	Forward	CCAGGACCAGGAAGCAACTTTAGG
	Reverse	CCTCCTCCATAACCAGGGCTACC
YTHDF2	Forward	TTGCCTCCACCTCCACCACAG
	Reverse	CCCATTATGACCGAACCCACTGC
OPA1	Forward	CGCTTCAAGGTCGTCTCAAGGATAC
	Reverse	CACTGCTCTTGGGTCCGATTCTTC
pre-miR-208a-5p	Forward	TGACGGGTGAGCTTTTGG
	Reverse	TTGCTCGTCTTATACGTGAGTG
mmu-miR-669a-3p		CGCGACATAACATACACACACACGT
mmu-miR-466q		AAGTTGCAGTGCACACACACA
mmu-miR-139-3p		TATATATGGAGACGCGGCCCTGTTG
mmu-miR-871-5p		CGCGTATTCAGATTAGTGCCAGTCATG
mmu-miR-208a-5p		TGAGCTTTTGGCCCGGGTTATAC

### m6A content analysis

m6A in total RNA was analyzed with the EpiQuik m6A RNA Methylation Quantification Kit as the manufacturer’s protocol (Epigentek, #P-9005). Total RNA samples of 200 ng were used to determine the percentage of m6A. Firstly, RNAs were bound to the assay wells. Then, the capture antibody, detection antibody and enhancer solution were added separately. Finally, adding color developing solution for color development and measuring absorbance. The m6A levels were quantified by reading the absorbance of each well at a wavelength of 450 nm, and calculations were performed based on the standard curve.

### Western blot analysis

MSFs and skin tissues were first washed with ice-cold PBS and subsequently lysed in a cell lysis buffer supplemented with a protease inhibitor cocktail (Cell Signaling Technology Inc.). The protein concentration in the lysates was determined using the bicinchoninic acid (BCA) Protein Assay Kit (Beyotime). For SDS-PAGE, 20 μg of protein from each sample was loaded per well and then transferred onto a nitrocellulose membrane (Bio-Rad). The membrane was blocked by incubation with 5% fat-free milk. It was then incubated overnight at 4°C with primary antibodies directed against METTL3 (Cell Signaling Technology, United States), p53 (Cell Signaling Technology, United States), p21 (Proteintech), OPA1 (Proteintech), and GAPDH (Proteintech). Following this, the blots were incubated with appropriate secondary antibodies. The immunoreactive bands were visualized using chemiluminescence with the Immobilon Western detection system (Millipore, United States).

### Dual-luciferase reporter assay

Cells were co-transfected with plasmids harboring the 3′-untranslated region (3′-UTR) of either wild-type or mutant fragments from the OPA1 gene, along with miRNA mimics, using Lipofectamine RNAiMAX (Invitrogen) in accordance with the manufacturer’s protocol. Forty-eight hours post-transfection, the activities of firefly and Renilla luciferase were measured sequentially using a dual-luciferase reporter assay system (BioTek Instruments, Inc.). Ultimately, the ratios of luminescence from firefly to Renilla luciferase were calculated to assess the impact of miRNA on OPA1 3′-UTR activity.

### Methylated RIP-qPCR

The riboMeRIP™ m6A Transcriptome Profiling Kit (RiboBio, Guangzhou, China) was utilized to examine m6A RNA modifications, following the manufacturer’s instructions. In summary, anti-m6A magnetic beads were prepared and used for immunoprecipitation, followed by RNA recovery. The recovered RNAs were then detected by qRT-PCR. The IP/Input ratio was calculated by 2^-ΔCt^ (ΔCt =Ct^IP^ - Ct^Input^) and corrected by the ratio of the IP RNA template and the Input RNA template to the start RNA when reverse transcription was introduced.

### Statistical analysis

All data were analyzed using SPSS 22.0 for normality testing and are presented as the mean ± SEM. Between-group differences were assessed via Student’s t-test (two groups) or ANOVA with *post hoc* tests (multiple groups). Statistical significance was defined as p < 0.05; non-significant differences are denoted as “ns.”

## Results

### Decreased METTL3 and m6A modification in senescent fibroblasts and aging skin tissue

**Figure 2:**
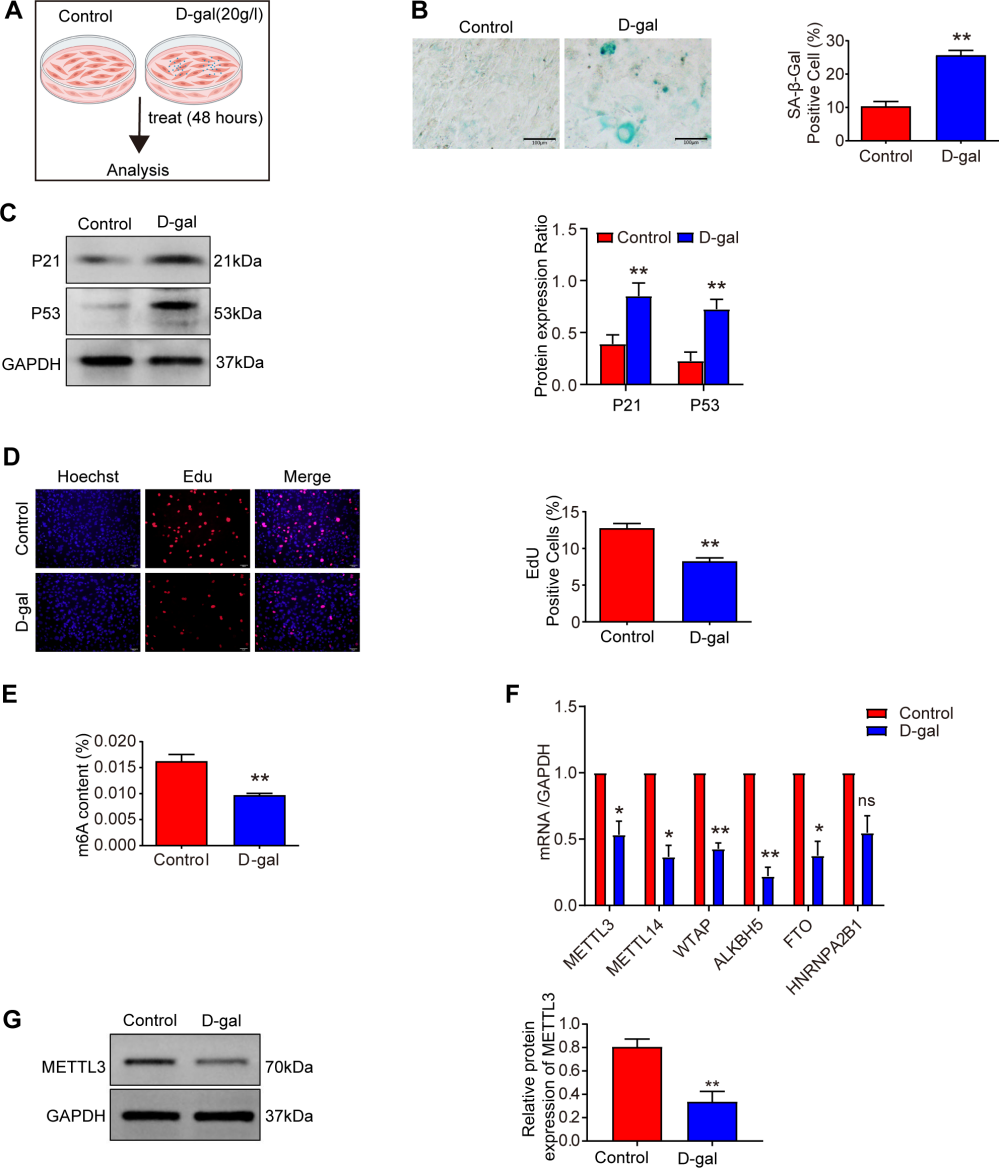
METTL3 and m6A modifications are decreased in senescence. **(A)** Experimental scheme. **(B)** The level of cellular senescence was assessed using SA-β-gal staining in the control and D-gal groups after treatment with 20 g/l D-galactose for 48 hours (n = 3). Scale bars: 100 μm. **(C)** Western blot analysis of P21 and P53 expression levels in D-galactose-induced MSFs (n = 3). **(D)** EdU incorporation assay assessing the proliferation of MSFs in control and D-gal groups after exposure to 20 g/L D-galactose for 48 hours (n = 3). Cells were stained with the nucleic acid dye Hoechst (blue) and EdU (red). Scale bar: 50 μm. **(E)** m6A modification levels were measured using an m6A quantification assay in D-galactose-induced senescent MSFs (n = 3). **(F)** Expression of m6A-modified mRNA was measured by qRT-PCR in D-galactose-induced senescent MSFs (n = 3). **(G)** Western blot analysis of METTL3 expression in D-galactose-induced senescent MSFs (n = 3). All data are presented as mean ± SEM. *p < 0.05, **p < 0.01, and ns indicates no significant difference. D-gal, D-galactose. **Figure 2A** was created with BioRender.com.

Cellular senescence is characterized by increased senescence-associated β-galactosidase (SA-β-gal) activity, elevated expression of p53 and p21, and histological changes in skin tissue, including a significant reduction in dermal thickness, indicative of age-related structural deterioration (25). In this study, we utilized D-galactose (D-gal)-induced senescence models in mouse skin fibroblasts (MSFs) and aging mouse models to explore uncharacterized aspects of m6A biology in aging. After 48 hours of D-gal treatment at a concentration of 20 g/L (**Figure 2A**), we observed a marked increase in SA-β-gal-positive cells (**Figure 2B**), elevated expression of senescence-related proteins p21 and p53 in MSFs (**Figure 2C**), and a significant reduction in the proliferative capacity of MSFs (**Figure 2D**). Additionally, the dermal layer was notably thinner in aging skin (**Figure 1B**), and p21 protein expression was upregulated in aged skin tissue (**Figure 1C**).

To investigate the relationship between senescence and m6A methylation, we first measured m6A levels in senescent MSFs and found a significant decline in m6A modification (**Figure 2E**). Subsequently, we performed a screen of m6A-related gene expression, including METTL3, METTL14, WTAP, ALKBH5, and HNRNPA2B1 (**Figures 2F**, **1D**). Among the methyltransferase genes (METTL3, METTL14, and WTAP), METTL3 expression was significantly downregulated in both aging skin and senescent MSFs. Consistently, METTL3 protein levels were also reduced in these aging models (**Figures 2G**, **1E**). These findings suggest that METTL3 deficiency may contribute to the loss of m6A during aging.

### METTL3 is indispensable for preventing MSFs from accelerated senescence

**Figure 3:**
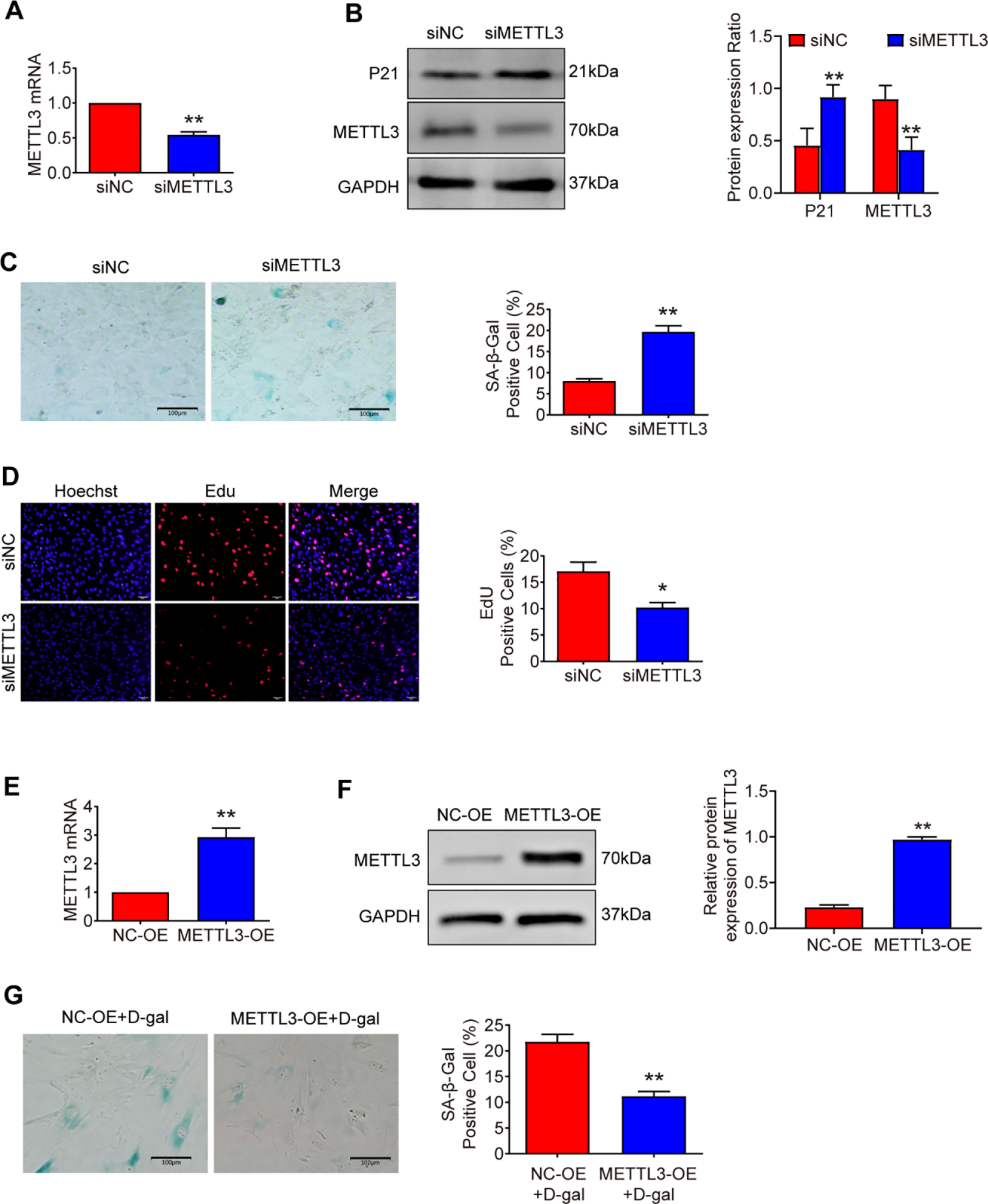
METTL3 is indispensable for preventing MSFs from accelerated senescence. **(A)** METTL3 mRNA expression in MSFs transfected with METTL3 siRNA for 48 hours (n = 3). **(B)** Western blot analysis of P21 and METTL3 expression levels in METTL3-deficient MSFs (n = 3). **(C)** The degree of cellular senescence was measured by SA-β-gal staining in METTL3-deficient MSFs (n = 3). Scale bars: 100 μm. **(D)** Proliferative capacity was assessed by EdU assay in METTL3-deficient MSFs (n = 3). Scale bars: 50 μm. **(E, F)** The efficiency of METTL3 overexpression was verified by qRT-PCR and Western blot analysis (n = 3). **(G)** The degree of cellular senescence was measured by SA-β-gal staining in METTL3-overexpressing MSFs after treatment with 20 g/L D-galactose for 48 hours (n = 3). Scale bars: 100 μm. All data are presented as mean ± SEM. *p < 0.05, **p < 0.01. siNC, negative control siRNA; siMETTL3, METTL3 siRNA; NC-OE, negative control overexpression; METTL3-OE, METTL3 overexpression.

Consistent with previous studies that have used loss - of - function strategies to investigate the roles of METTL3 (19), we designed siRNAs to specifically silence METTL3 expression in mouse skin fibroblasts (MSFs). This intervention led to a significant reduction in METTL3 expression compared to control conditions (**Figures 3A, B**). Furthermore, MSFs with METTL3 deficiency exhibited senescent phenotypes, characterized by increased expression of the aging marker protein p21 (**Figure 3B**), an increased percentage of cells positive for SA - β - Gal staining (**Figure 3C**), and diminished proliferative capacity (**Figure 3D**). Collectively, these results indicate that METTL3 deficiency accelerates the onset of senescence in MSFs.

We then overexpressed METTL3 in MSFs (**Figures 3E, F**). As anticipated, the overexpression of METTL3 was able to rescue the senescence induced by D - galactose (**Figure 3G**). Together, these findings suggest that METTL3 plays a crucial role in delaying senescence in MSFs.

### METTL3-YTHDF2-dependent m6A methylation promotes miR-208a-5p decay

**Figure 4:**
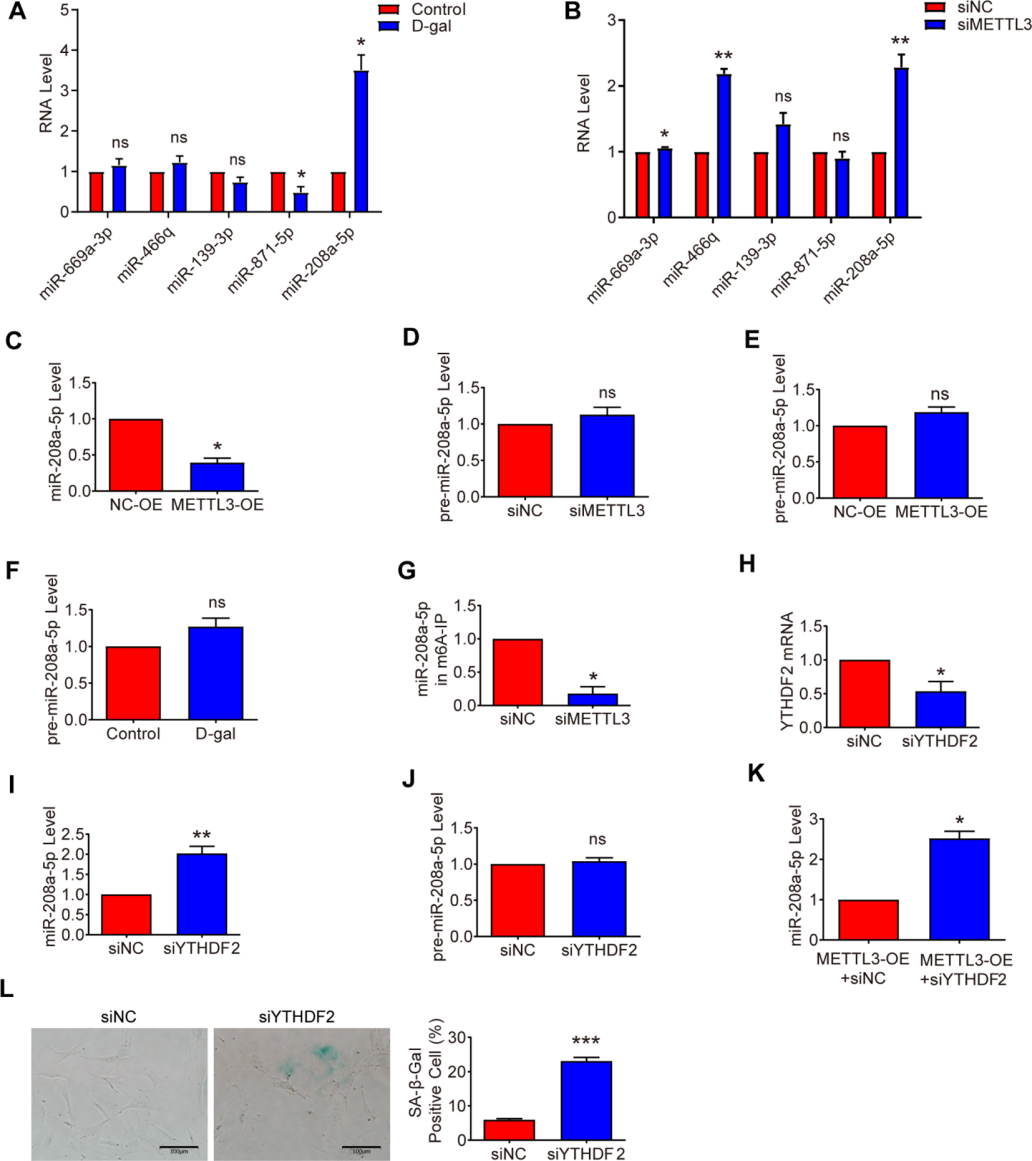
METTL3-YTHDF2-dependent m6A methylation promotes the decay of miR-208a-5p. **(A, B)** miRNA expression was measured by qRT-PCR in senescent and METTL3-deficient MSFs (n = 3). **(C)** miR-208a-5p expression was measured by qRT-PCR in METTL3-overexpressing MSFs (n = 3). **(D–F)** pre-miR-208a-5p expression was measured by qRT-PCR in METTL3-deficient, METTL3-overexpressing, and D-galactose-induced senescent MSFs (n = 3). **(G)** Inhibitory effect of METTL3 siRNA (siMETTL3) on miR-208a-5p methylation by m6A relative to that of negative control siRNA (siNC) (n = 3). **(H–J)** YTHDF2 mRNA, miR-208a-5p, and pre-miR-208a-5p expression was measured by qRT-PCR in MSFs transduced with YTHDF2 siRNA for 48 hours (n = 3). **(K)** miR-208a-5p expression was measured by qRT-PCR in the METTL3-overexpression + negative control siRNA (METTL3-OE+siNC) group and the METTL3-overexpression + YTHDF2 siRNA (METTL3-OE+siYTHDF2) group (n = 3). **(L)** The degree of cellular senescence was measured by SA-β-gal staining in YTHDF2-deficient MSFs (n = 3). Scale bars: 100 μm. All data are presented as mean ± SEM. *p < 0.05, **p < 0.01, ***p < 0.001, and ns indicates no significant difference. siYTHDF2: YTHDF2 siRNA.

Analysis of aging skin microarray data (21) revealed differential expression of miR-669a-3p, miR-466q, miR-139-3p, miR-871-5p, and miR-208a-5p in aged tissues. We subsequently examined the expression of these five miRNAs in aging and METTL3-deficient MSFs. Among them, miR-208a-5p was significantly upregulated (>3-fold) in D-gal-induced senescent MSFs (**Figure 4A**) and exhibited a dramatic increase in aging skin (**Figure 1F**). Additionally, miR-208a-5p expression was significantly elevated in METTL3-deficient MSFs (**Figure 4B**), while METTL3 overexpression had the opposite effect (**Figure 4C**). Interestingly, pre-miR-208a-5p levels remained unchanged across METTL3-deficient, METTL3-overexpressing, or D-gal-induced senescent MSFs (**Figures 4D–F**), suggesting that METTL3 regulates fibroblast senescence by modulating miR-208a-5p expression.

Next, we confirmed the effects of METTL3 deficiency on the m6A modification levels of miR - 208a - 5p using m6A qPCR. As shown in **Figure 4G**, miR - 208a - 5p was significantly less enriched after METTL3 inhibition, indicating that miR - 208a - 5p is a target of METTL3 in MSFs. Given that METTL3 - mediated m6A methylation reduces miR - 208a - 5p expression, we hypothesized that miR - 208a - 5p is a target of YTHDF2, which promotes the decay of m6A - methylated RNAs (28). Consistent with our hypothesis, the level of miR - 208a - 5p significantly increased upon YTHDF2 silencing, while the level of pre - miR - 208a - 5p did not show a significant difference (**Figures 4H–J**). Moreover, co - transfection with siRNA targeting YTHDF2 and an overexpression plasmid for METTL3 in MSFs led to increased expression of miR - 208a - 5p (**Figure 4K**). Interestingly, an increase in SA - β - Gal - positive cells was observed in YTHDF2 - deficient MSFs (**Figure 4L**). Collectively, these findings indicate that METTL3 promotes the decay of miR - 208a - 5p in a YTHDF2 - dependent manner, consistent with previous studies showing that YTHDF2 can regulate the expression of miRNAs by binding to m6A - modified sites and promoting their decay.

### miR-208a-5p inhibitor rescues MSFs senescence induced by silence of METTL3

**Figure 5:**
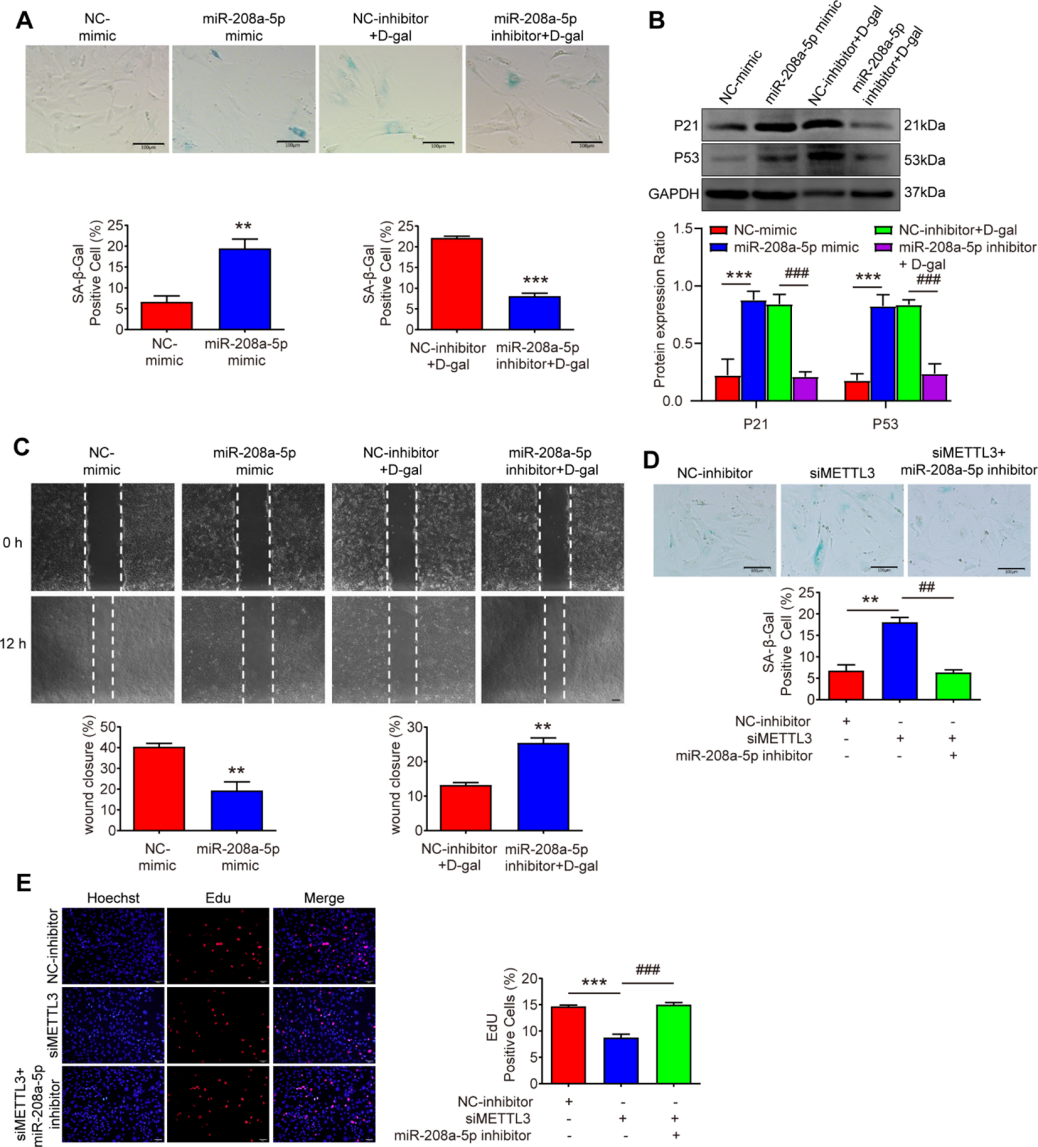
miR-208a-5p inhibitor rescues MSFs senescence induced by the silence of METTL3. **(A)** Cellular senescence was assessed via SA-β-gal staining in MSFs transfected with miR-208a-5p mimic for 48 h or co-treated with D-gal and miR-208a-5p inhibitor for 48 h (n=3). Scale bars: 100μm. **p < 0.01, ***p < 0.001. **(B)** Western blot analysis of P21 and P53 protein expression levels (n=3). **p < 0.01 vs. NC-mimic; ##p < 0.01 vs. NC-inhibitor + D-gal. **(C)** Scratch wound healing assay demonstrating miR-208a-5p’s effect on cell migration. Wound closure was quantified 12 h post-scratch (n=3). Scale bars, 100μm. **p < 0.01. **(D)** SA-β-gal staining showing senescence levels in MSFs co-transfected with METTL3 siRNA and miR-208a-5p inhibitor (n=3). Scale bars: 100μm. **p < 0.01 vs. NC-inhibitor; ##p < 0.01 vs. siMETTL3. **(E)** Proliferative capacity evaluated by EdU incorporation assay (n=3). Scale bars: 50μm. ***p < 0.001 vs. NC-inhibitor; ###p < 0.001 vs. siMETTL3. NC-mimic, negative control mimic; NC-inhibitor, negative control inhibitor.

To investigate the role of miR - 208a - 5p in MSFs, we introduced miR - 208a - 5p mimics and inhibitors. After treatment with the miR - 208a - 5p mimic for 48 hours, MSFs exhibited aging phenotypes, characterized by an increased percentage of cells positive for SA - β - Gal staining (**Figure 5A**), reduced migration capacity (**Figure 5C**), and elevated expression of the senescence markers p21 and p53 (**Figure 5B**). Conversely, treatment with the miR - 208a - 5p inhibitor produced opposing effects (**Figures 5A–C**). These results confirm that overexpression of miR - 208a - 5p promotes senescence in MSFs.

Subsequently, we introduced the corresponding miRNA inhibitor into METTL3 - deficient MSFs. The miR - 208a - 5p inhibitor significantly counteracted the senescence - promoting effects of METTL3 silencing (**Figure 5D**). Moreover, the inhibitor largely restored the proliferative capacity of the cells (**Figure 5E**). Thus, the data indicate that the miR - 208a - 5p inhibitor can reverse the senescence associated with METTL3 deficiency, suggesting that reduced METTL3 expression leads to increased miR - 208a - 5p levels, which in turn contribute to the senescence of MSFs.

### miR-208a-5p promotes senescence by targeting OPA1

**Figure 6:**
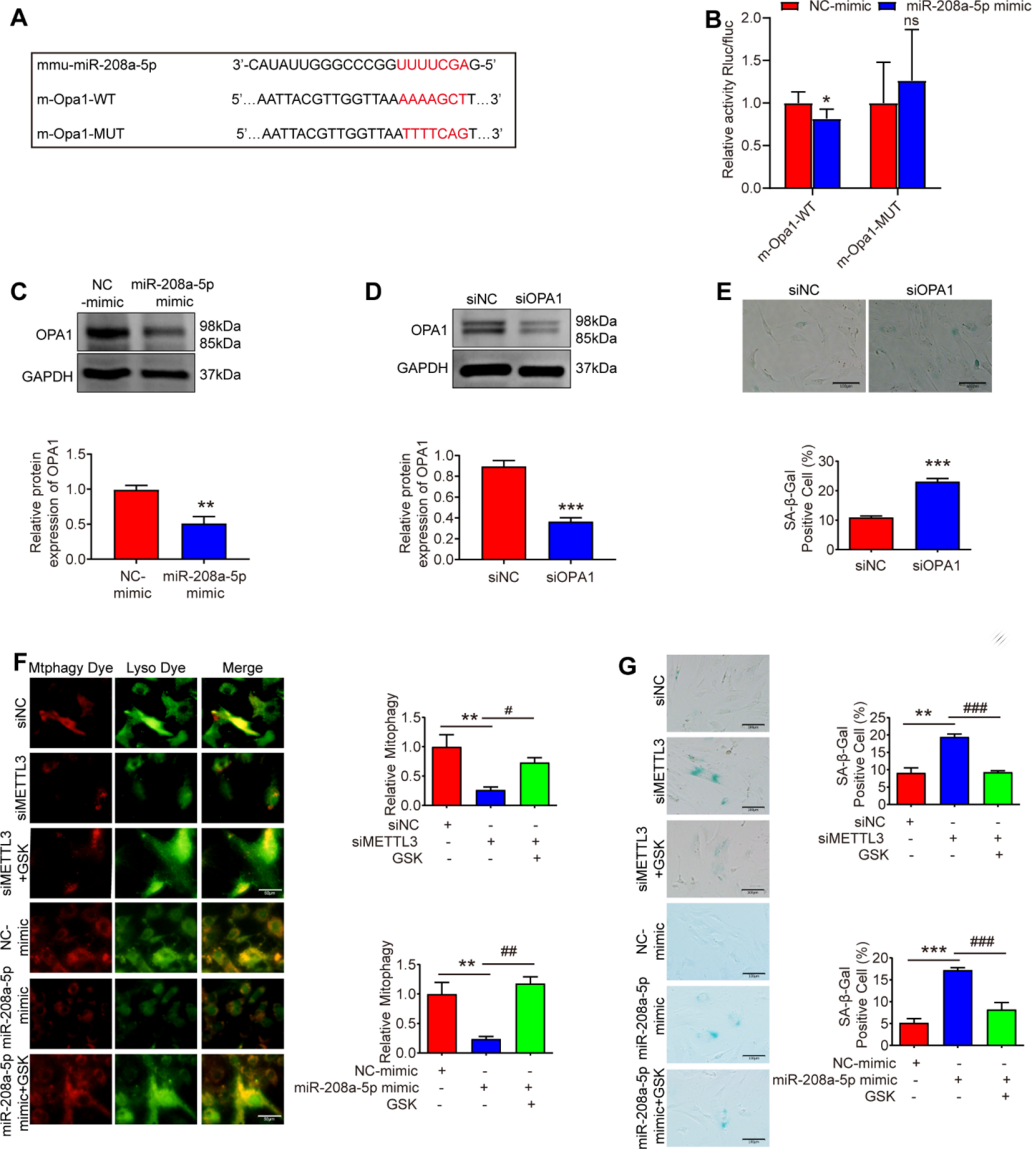
miR-208a-5p promotes senescence by targeting OPA1. **(A)** Predicted binding sites of miR-208a-5p on the OPA1 mRNA. **(B)** A dual luciferase reporter assay confirmed the targeted interaction between OPA1 and miR-208a-5p (n=5). *p < 0.05; ns indicates statistically insignificant. **(C)** Western blot analysis revealed reduced OPA1 expression 48 hours post-transfection with miR-208a-5p mimic (n=3), **p < 0.01. **(D)** OPA1 knockdown via siRNA significantly decreased its expression, as shown by Western blot 48 hours post-transfection (n=3), **p < 0.01. **(E)** SA-β-gal staining quantified cellular senescence in OPA1-deficient MSFs. Scale bars: 100 μm, ***p < 0.001. **(F)** Mitophagy levels were assessed using a Mitophagy Detection Kit in siNC, siMETTL3, and siMETTL3 + GSK2578215A (GSK) groups. Scale bars: 50μm; *p < 0.05 vs. siNC, ##p < 0.01 vs. siMETTL3; *p < 0.05 vs. NC-mimic, ##p < 0.01 vs. miR-208a-5p mimic. **(G)** Degree of cellular senescence was measured by SA-β-gal staining (n=3). Scale bars: 100 μm, **p < 0.01 vs. siNC, ###p< 0.001 vs. siMETTL3; ***p < 0.001 vs. NC-mimic, ###p< 0.001 vs. miR-208a-5p mimic. GSK, GSK2578215A.

Using TargetScan (http://targetscan.org/), we predicted the potential interaction between miR - 208a - 5p and the 3′ - UTR of OPA1 mRNA (**Figure 6A**). In the dual - luciferase assay (**Figure 6B**), cotransfection of the wild - type (WT) OPA1 3′ - UTR with the miR - 208a - 5p mimic significantly reduced luciferase activity compared to the control group. In contrast, the miR - 208a - 5p mimic did not affect luciferase expression in cells transfected with the mutant OPA1 3′ - UTR. Moreover, OPA1 expression can be modulated by miR - 208a - 5p (**Figure 6C**). OPA1, located in the inner mitochondrial membrane, regulates mitochondrial fusion. Downregulation of OPA1 is associated with the inhibition of mitophagy (29, 30). Mitophagy is the process by which cells remove damaged or dysfunctional mitochondria through autophagolysosomes, thereby maintaining the homeostasis of mitochondrial number and quality within cells (31). To examine the role of OPA1 in MSFs, we developed siRNAs to silence OPA1 in MSFs (**Figure 6D**). After treatment with OPA1 siRNA, MSFs exhibited aging phenotypes, characterized by an increased percentage of cells positive for SA - β - Gal staining (**Figure 6E**). Interestingly, the mRNA levels of OPA1 were found to be reduced in aging skin (**Figure 1G**).

To evaluate the roles of METTL3 and miR - 208a - 5p in mitophagy, we analyzed the impact of silencing METTL3 and overexpressing miR - 208a - 5p on this process. The results demonstrated that both the silencing of METTL3 and the overexpression of miR - 208a - 5p led to a reduction in mitophagy (**Figure 6F**). However, treatment with GSK (a mitophagy inducer) for 24 hours (27) was able to rescue both mitophagy and senescence (**Figures 6F, G**). These findings suggest that miR - 208a - 5p can promote senescence by targeting OPA1, thereby modulating mitophagy.

## Discussion

As a pivotal RNA modification in higher eukaryotes, N6-methyladenosine (m6A) has emerged as a critical epigenetic regulator of diverse biological processes, including aging and age-related pathologies (32, 33). While recent studies implicate m6A dysregulation in premature aging of mesenchymal stem cells (19) and FTO-mediated ovarian aging (34), its role in cutaneous senescence remains poorly characterized. Our investigation reveals diminished METTL3 expression in senescent dermal fibroblasts, with functional studies demonstrating that METTL3 silencing induces cellular senescence while its overexpression delays senescent phenotypes.

Mechanistically, METTL3 depletion disrupts DGCR8-pri-miRNA interactions, impairing global miRNA maturation (35). In bladder cancer and cardiac hypertrophy models, METTL3 exerts oncogenic and pro-fibrotic effects by facilitating DGCR8-mediated maturation of pri-miR-221/222 in an m6A-dependent manner (35, 36). Conversely, in hypertrophic scars, METTL3 overexpression drives pathological fibrosis through m6A modification of pri-miR-31. This modification recruits the m6A reader YTHDF2, destabilizing the precursor and suppressing mature miR-31-5p biogenesis, thereby de-repressing ZBTB20 to promote collagen overproduction. While METTL3 typically enhances miRNA biogenesis, such as upregulating miR-320 via pre-miR-320 stabilization (37), our data reveal a context-dependent exception: METTL3 inhibition selectively elevates mature miR-208a-5p without altering pre-miR-208a-5p levels. This suggests a unique regulatory mechanism that operates independently of canonical DGCR8-dependent processing. These findings underscore METTL3’s dual functionality, where its role in miRNA regulation is dictated by disease-specific m6A site localization and reader protein engagement. Structural analysis of miR-208a-5p (5’-GAGCUUUUGGCCCGGGUUAUAC-3’) reveals conserved [A/U][A/C/G]A[C/G][A/C] motifs at its termini, consistent with predicted m6A modification sites (38). YTHDF2, a key m6A reader protein, drives the degradation of m6A-modified RNAs (39–41). Our findings show that YTHDF2 knockdown abolished the miR-208a-5p suppression caused by METTL3 overexpression. Leveraging the established role of YTHDF2 in RNA degradation (41), our findings delineate a METTL3/YTHDF2 axis that governs miR-208a-5p turnover during fibroblast senescence.

The significance of miR-208a-5p in aging is highlighted by its notable upregulation in senescent skin fibroblasts and its pro-senescence effects when overexpressed, which is consistent with previous reports that miR-208a-5p can inhibit cell migration by targeting DAAM1 (42). Importantly, the senescence induced by METTL3 siRNA can be reversed by inhibiting miR-208a-5p, and mechanistic studies have identified OPA1 as a direct downstream target of miR-208a-5p. This reveals a functional link between METTL3-mediated m6A modification and miRNA activity in the context of aging. Furthermore, this regulatory axis has been shown to play a conserved role across different tissues. For example, in human renal tubular epithelial cells, the loss of METTL3 activates NF-κB-driven senescence, which can be counteracted by the METTL3/miR-181a-5p/IL-1α pathway (43). Similarly, in diabetic retinopathy, METTL3 deficiency exacerbates RPE apoptosis under hyperglycemic conditions, an effect that can be alleviated through miR-25-3p/PTEN/Akt signaling (44). These examples illustrate that despite tissue-specific differences, METTL3 acts as a central regulator of senescence by modulating diverse downstream targets such as OPA1, NF-κB, and PTEN via its m6A-dependent regulation of miRNAs (miR-208a-5p, miR-181a-5p, miR-25-3p), thereby controlling mitochondrial integrity, inflammation, and cell survival. While therapeutic targeting of this axis holds promise for mitigating age-related pathologies, tissue-specific delivery systems must be optimized to address divergent miRNA effector networks.

Our findings highlight the critical role of mitochondrial quality control in senescence regulation. Notably, both miR-208a-5p overexpression and METTL3 depletion significantly impaired mitophagy, leading to the accumulation of dysfunctional mitochondria. This mitochondrial compromise resulted in elevated reactive oxygen species (ROS) production, which triggered DNA damage and cellular stress—key hallmarks of senescence (45, 46). Pharmacological induction of mitophagy using GSK effectively rescued mitochondrial dysfunction by enhancing the clearance of damaged mitochondria, restoring both mitochondrial mass and functional integrity (47). Critically, this restoration of mitochondrial homeostasis reduced ROS overproduction and mitigated cellular stress, ultimately attenuating senescence progression. This positions miR-208a-5p within an emerging class of senescence-associated miRNAs regulating mitophagy, including miR-30a/BNIP3L (48), miR-181a (49), and miR-155/PINK1 (50) pathways. Our data establish a novel METTL3/miR-208a-5p/OPA1 axis where m6A deficiency drives miR-208a-5p accumulation, impairing mitochondrial quality control and accelerating fibroblast senescence.

Our study outlines the METTL3-m6A-YTHDF2 axis in fibroblast senescence, but some limitations exist. First, *in vivo* validation of this axis via tissuespecific YTHDF2 knockout or METTL3 transgenic models is still needed to boost translational relevance. Second, while inhibiting miR-208a-5p or overexpressing OPA1 rescued senescence *in vitro*, *in vivo* interventions are required to verify their therapeutic potential. Third, resource limits prevented us from conducting large-scale omics analyses to map out broader m6A-regulated networks. Finally, sex-specific differences in METTL3/miR-208a-5p signaling remain unexplored, potentially impacting mechanistic generalizability. Future research using conditional models, *in vivo* rescue assays, multi omics methods and sex-stratified analyses will boost the development of anti-aging strategies using epitranscriptomic regulation.

## Conclusions

This study elucidates a previously unrecognized epitranscriptomic circuit linking m6A metabolism to cutaneous aging through mitophagic regulation. The METTL3/YTHDF2/miR-208a-5p/OPA1 axis provides mechanistic insights into skin fibroblast senescence and identifies potential therapeutic targets for combating age-related dermatological disorders.

## Data Availability

The original contributions presented in the study are included in the article/supplementary material. Further inquiries can be directed to the corresponding authors.
